# Recovery from diabetes mellitus in primary aldosteronism patients after adrenalectomy

**DOI:** 10.1186/s12902-022-01254-6

**Published:** 2022-12-27

**Authors:** Yu Liu, Lede Lin, Chi Yuan, Sikui Shen, Yin Tang, Zhihong Liu, Yuchun Zhu, Liang Zhou

**Affiliations:** 1Department of Urology, Institute of Urology (Laboratory of Reconstructive Urology), West China Hospital, Sichuan University, No. 37 Guo Xue Xiang, 610041 Chengdu, Sichuan P.R. China

**Keywords:** Adrenalectomy, Diabetes mellitus, Primary aldosteronism, Remission, Improvement, Risk factors

## Abstract

**Background:**

The prevalence of diabetes mellitus (DM) was higher in primary aldosteronism (PA) patients. We aimed to evaluate the outcome of DM after adrenalectomy and determine the factors associated with that in PA patients.

**Methods:**

PA patients with DM (PA + DM patients) who received adrenalectomy were recruited into the study. The patients were classified into 3 groups based on their DM conditions after treatment, including “remission”, “improved” and “unchanged” groups. Univariate and multivariate logistic regression analysis was conducted to uncover the preoperative factors affecting the outcome of DM after adrenalectomy.

**Results:**

A total of 54 PA + DM patients received adrenalectomy. After adrenalectomy, 16.7%, 33.3% and 50.0% of patients were classified into the “remission”, “improved” and “unchanged” groups, respectively. The factors negatively associated with remission or improvement from DM after adrenalectomy were longer duration of hypertension (*P* = 0.029). Higher concentration of urinary magnesium (*P* = 0.031) and higher 24 h urinary potassium (*P* = 0.049) were factors negatively associated with the “remission” from DM after adrenalectomy.

**Conclusions:**

Adrenalectomy was beneficial for the remission and improvement from DM in the half of PA patients with DM. Longer duration of hypertension, higher concentration of urinary magnesium and higher 24 h urinary potassium may prevent the remission and improvement from DM after adrenalectomy in PA patients. Examination of urinary electrolyte could be considered in PA patients with DM for predicting the outcome of DM after adrenalectomy.

**Supplementary Information:**

The online version contains supplementary material available at 10.1186/s12902-022-01254-6.

## Introduction

Primary aldosteronism (PA) is characterized by hypertension, excessive aldosterone excretion and low serum potassium. Its prevalence in hypertensive populations ranges from 4–10% [1]. Adrenalectomy and mineralocorticoid receptor antagonist are the recommended therapies for PA [2]. The prevalence of diabetes mellitus (DM) is higher in PA patients (21.6%) compared to that in essential hypertension patients (14.3%) [3]. Excessive plasma aldosterone [4] or coexisting high cortisol secretion [5, 6] may reduce insulin secretion and sensitivity, leading to DM. A recent interesting study showed that adrenalectomy would improve insulin secretion in PA patients [4]. In addition, DM was less likely to occur in PA patients after adrenalectomy than in essential hypertension patients [7]. However, there were also some studies reporting that blood glucose did not decrease after adrenalectomy [4, 8–10]. Thus, it was still unclear whether adrenalectomy could improve blood glucose metabolism and DM conditions. We hypothesized that adrenalectomy only improved the glucose metabolism in a part of PA patients. We previously reviewed the PA patients in our institute and found several new factors affecting DM in this population, such as higher blood urea nitrogen and higher urinary calcium [11]. The present study evaluated the outcome of DM after adrenalectomy and determined the clinical factors affecting the remission and improvement from DM after adrenalectomy in PA patients with DM (PA + DM patients).

## Methods

### Study design

This retrospective study was conducted following the Strengthening the Reporting of Observational Studies in Epidemiology guideline [12]. The West China Hospital of Sichuan University Medical Research Ethics Committee approved this study (IRB approval number 2,019,568). The study was performed according to the Declaration of Helsinki of 1964 and its later amendments.

### Study population

We selected PA patients with DM who received adrenal venous sampling from January 2018 to January 2021 at our institute. PA was diagnosed using the American Endocrine Society guideline [2]. Patients who had symptoms, such as dizziness, headache, palpitations, weakness or acroanesthesia, would receive examination of blood pressure, blood biochemistry and blood hormones involved in the renin-angiotensin-aldosterone system. Potential PA patients were identified by blood aldosterone-to-renin ratio (ARR) ≥ 30. Then, these patients received saline infusion tests and captopril tests to verify the diagnosis of PA. We also performed the adrenal venous sampling with adrenocorticotropic hormone stimulus to determine the dominant side. Successful cannulation of the adrenal veins was confirmed by a selectivity index ≥ 2. The dominant aldosterone-producing side was determined using a lateralization index ≥ 2 [13]. DM was diagnosed according to the clinical guideline for DM published by the American Diabetes Association [14].

### Data collection

Demographics, medical history information, and preoperative laboratory test results were extracted from the medical records of PA patients with DM. Demographic factors included body mass index (BMI), gender and age. Medical history information included the maximal systolic blood pressure, duration of hypertension and DM, side and size of tumor and clinical symptoms. The results of laboratory tests before adrenalectomy were also collected, including renin-angiotensin-aldosterone system factors, urinary and blood electrolytes, factors of glucocorticoid metabolism, routine blood tests, hepatic and renal function factors, lipids and glucose metabolism indices. Fasting blood glucose and glycosylated hemoglobin (HbA1C) before surgery were also examined. Biochemical examinations were performed with a Roche Cobas 8000. Renin and aldosterone were examined with radioimmunoassay. Briefly, serum was incubated with a solid phase anti-aldosterone or anti-renin monoclonal antibody at 37℃ for 1 h. Then, unbound material was removed. The concentrations of renin and aldosterone were calculated according to standard curves. The intra-assay coefficient of variation (CV) was < 10.4%, and the inter-assay CV was < 15.6%.

### Follow-ups

PA patients with DM were followed up to obtain DM and hypertension conditions one year after adrenalectomy. They also received examinations of fasting blood glucose and HbA1C. First, we separated the patients into 3 groups according to the use of antidiabetic medications. Patients in the “remission” group were not taking antidiabetic medications any longer and the fasting blood glucose and HbA1C were within the normal range. The following conditions were considered as “improved”. First, patients had fewer types of antidiabetic medications than before or fewer doses of medications (without any change of types of medications), while the fasting blood glucose or HbA1C was the same or lower than before. Second, the types of antidiabetic medications did not change, but the fasting blood glucose or HbA1C was within the normal range after treatment. The remaining patients were classified as “unchanged”. Similarly, they were also divided into three groups according to their antihypertensive medications after treatment. The “remission”, “improved” and “unchanged” groups included patients having no, less, and the same or more medications to maintain blood pressure within a normal range after treatment, respectively.

### Statistical analysis

Categorical variables were presented as quantity (percentage) and were analyzed using Fisher’s exact test or Chi-square test. Quantitative variables were reported as the mean (standard deviation) if they were symmetrically distributed; otherwise, they were presented as median (interquartile range). Quantitative variables were analyzed by the Kruskal-Wallis test or analysis of variance (ANOVA) between three groups and by Mann-Whitney test or Student’s t-test between two groups. Univariate and multivariate logistic regression analyses were conducted to identify the parameters affecting the remission and improvement from DM. Statistical analyses were conducted using AKIBM SPSS Statistics 24.0 (IBM Corp., Armonk, NY, USA). *P* values < 0.05 was considered significantly different.

## Results

### Study participants

In all, 321 PA patients received adrenal venous sampling at our institute. Among them, 63 (19.6%) PA patients had DM, and 54 (85.7%) of them were available for follow-ups (Table 1). The mean age was 56.65 ± 10.31 years, and the mean BMI was 26.35 ± 3.05 kg/m^2^. The number of males (*n* = 34) accounted for 63% of all the PA patients with DM. One year after adrenalectomy, 9 (16.7%) “remission” patients did not need antidiabetic medications any longer, and their fasting blood glucose and HbA1C levels were within the normal ranges; 18 (33.3%) “improved” patients had lower fasting blood glucose or HbA1C levels after surgery, while they needed fewer types or doses of antidiabetic medications. The remaining 27 (50.0%) “unchanged” patients maintained the same therapy for DM as before and their fasting blood glucose and HbA1C levels did not decrease after surgery (Supplementary Table 1). Similarly, with regard to hypertensive conditions, the “remission”, “improved” and “unchanged” groups had 17 (31.5%), 31 (57.4%) and 6 (11.1%) patients, respectively. There was no correlation between the remission or improvement from hypertension and that from DM (*P* = 0.428). After adrenalectomy, both the median fasting blood glucose (6.36 vs. 7.55 mmol/L, *P* = 0.001) and median HbA1C (6.40 vs. 6.60, *P* = 0.002) levels were lower than those before surgery. The median fasting blood glucose (*P* = 0.398) and median HbA1C (*P* = 0.068) before surgery were not significantly different between three groups. However, the median fasting blood glucose was lower in the “remission” group (5.23 mmol/L), followed by the “improved” group (6.15 mmol/L) and “unchanged” (7.08 mmol/L) group after surgery (*P* = 0.001). Median HbA1C was also lower in the “remission” group (5.70%) than in the “improved” group (6.45%) and “unchanged” group (6.50%) (*P* = 0.001). After surgery, the mean serum potassium levels of all patients were higher than 3.5 mmol/L. All the patients’ median plasma aldosterone levels (lying or standing position) and median ARRs dropped below the upper limit of normal.

**Table 1:** Baseline characteristics of the patients with PA and DM receiving adrenalectomy

	Factors	Overall (*n* = 54)	Remission (*n* = 9)	Improved (*n* = 18)	Unchanged (*n* = 27)	*P* value
**Clinical characteristics**	Age (years)	56.65 (10.31)	46.56 (8.35)	59.56 (9.38)	57.74 (10.10)	0.010^*^
	Gender (female/male)	20 (37.0)/34 (63.0)	2 (22.2)/7(77.8)	8 (44.4)/10(55.6)	10 (37.0)/17(63.0)	0.530^‡^
	BMI (kg/m^2^)	26.35 (3.05)	25.94 (3.26)	26.67 (2.72)	26.28 (3.28)	0.835^*^
	Results of hypertension (remission/improved/unchanged)	17 (31.5)/31 (57.4)/6 (11.1)	4 (44.4)/5 (55.6)/0 (0)	5 (27.8)/12 (66.7)/1 (5.6)	8 (29.6)/14 (51.9)/5 (18.5)	0.428^‡^
	Duration of hypertension (months)	102.00 (123.00)	96.00 (90.00)	84.00 (163.00)	120.00 (128.00)	0.202^†^
	Maximal SBP (mm Hg)	175.75 (34.66)	177.56 (40.62)	173.94 (41.64)	176.29 (28.70)	0.963^*^
	Duration of DM (months)	54.00 (88.75)	12.00 (20.00)	54.00 (92.00)	72.00 (92.00)	0.066^†^
	Order of hypertension and DM (hypertension-DM/DM-hypertension)	37 (68.5)/17 (31.5)	7 (77.8)/2(22.2)	14 (77.8)/4(22.2)	16 (59.3)/11(40.7)	0.342^‡^
	Maximal diameter of tumor (cm)	1.50 (0.60)	1.50 (0.90)	1.50 (0.82)	1.35 (0.55)	0.312^*^
	Dizziness or headache (no/yes)	29 (53.7)/25 (46.3)	6 (66.7)/9 (33.3)	8 (44.4)/10 (55.6)	15 (55.6)/12 (44.4)	0.531^‡^
	Palpitate (no/yes)	41 (75.9)/13 (24.1)	8 (88.9)/1 (11.1)	12 (66.7)/6 (33.3)	21 (77.8)/6 (22.2)	0.423^‡^
	Weakness or acroanesthesia (no/yes)	24 (44.4)/30 (55.6)	4 (44.4)/5 (55.6)	9 (50.0)/9 (50.0)	11 (40.7)/16 (59.3)	0.781^‡^
**Renin-angiotensin-aldosterone system factors**	ALD (lying position) (ng/dL)	23.22 (15.02)	28.18 (23.14)	19.50 (10.55)	23.82 (17.58)	0.343^†^
	ALD (standing position) (ng/dL)	27.59 (17.30)	27.42 (16.04)	28.08 (19.79)	27.59 (14.91)	0.214^†^
	ARR (lying position)	148.88 (272.10)	103.95 (664.30)	124.71 (127.71)	272.04 (540.23)	0.309^†^
	ARR (standing position)	87.67 (240.63)	87.67 (479.28)	61.83 (223.49)	110.84 (181.49)	0.815^†^
	PRA (lying position) (ng/ml·h)	0.11 (0.16)	0.12 (0.22)	0.14 (0.11)	0.10 (0.14)	0.289^†^
	PRA (standing position) (ng/ml·h)	0.32 (0.57)	0.33 (0.84)	0.39 (0.81)	0.28 (0.43)	0.779^†^
	AT2 (lying position) (ng/L)	52.53 (15.77)	50.60 (14.60)	55.36 (12.60)	51.32 (18.45)	0.719^*^
	AT2 (standing position) (ng/L)	57.79 (15.14)	61.85 (16.79)	59.83 (13.16)	54.05 (15.95)	0.355^*^
**Serum electrolytes**	Serum Na (mmol/L)	143.23 (2.61)	142.84 (2.27)	143.87 (2.95)	142.94 (2.49)	0.453^*^
	Serum K (mmol/L)	2.82 (0.61)	2.88 (0.58)	2.95 (0.60)	2.70 (0.63)	0.391^*^
	Serum Cl (mmol/L)	102.81 (2.92)	102.54 (2.72)	103.41 (2.14)	102.50 (3.42)	0.573^*^
	Serum Ca (mmol/L)	2.27 (0.11)	2.25 (0.11)	2.26 (0.10)	2.27 (0.12)	0.860^*^
	Serum Mg (mmol/L)	0.85 (0.09)	0.88 (0.06)	0.84 (0.08)	0.85 (0.10)	0.577^*^
	Serum P (mmol/L)	1.00 (0.21)	1.10 (0.13)	0.95 (0.18)	0.99 (0.25)	0.248^*^
**Urinary electrolytes**	Concentration of urinary K (mmol/L)	29.33 (12.48)	21.35 (13.92)	30.50 (13.79)	31.26 (10.41)	0.231^*^
	Concentration of urinary Na (mmol/L)	78.74 (39.50)	86.98 (60.34)	91.37 (35.59)	66.17 (31.65)	0.194^*^
	Concentration of urinary Cl (mmol/L)	77.63 (37.98)	76.08 (53.72)	92.84 (37.20)	66.54 (29.94)	0.172^*^
	Concentration of urinary Ca (mmol/L)	3.06 (2.59)	3.06 (0.88)	3.35 (4.00)	3.62 (3.32)	0.695^†^
	Concentration of urinary Mg (mmol/L)	2.32 (1.02)	1.66 (0.57)	2.63 (1.48)	2.43 (0.78)	0.187^*^
	Concentration of urinary P (mmol/L)	11.62 (5.02)	12.88 (6.62)	11.56 (6.16)	11.15 (3.85)	0.785^*^
	24 h urinary K (mmol/24 h)	59.99 (23.79)	35.45 (9.32)	58.15 (22.30)	65.99 (25.14)	0.085^*^
	24 h urinary Na (mmol/24 h)	170.56 (85.36)	123.33 (62.54)	195.21 (102.72)	158.61 (63.23)	0.293^*^
	24 h urinary Cl (mmol/24 h)	166.18 (86.24)	118.33 (55.39)	194.87 (106.91)	149.68 (56.21)	0.221^*^
	24 h urinary Ca (mmol/24 h)	6.95 (2.04)	5.94 (1.90)	6.91 (2.03)	7.39 (2.14)	0.507^*^
	24 h urinary Mg (mmol/24 h)	4.29 (1.29)	3.28 (1.22)	4.19 (1.27)	4.78 (1.19)	0.139^*^
	24 h urinary P (mmol/24 h)	21.57 (7.09)	19.24 (3.39)	18.64 (4.67)	24.85 (8.29)	0.138^*^
**Glucocorticoid metabolism**	PTC8 (Cortisol) (nmol/L)	349.04 (177.08)	313.57 (157.81)	400.41 (184.03)	326.96 (180.03)	0.445^*^
	PTC0 (Cortisol) (nmol/L)	77.64 (75.00)	44.77 (49.00)	91.71 (158.00)	82.61 (61.00)	0.146^†^
	ACTH (ng/L)	19.98 (19.00)	27.09 (41.00)	23.36 (15.11)	17.18 (39.00)	0.974^†^
	Total 24 h UFC (µg/24 h)	80.15 (84.92)	70.90 (90.10)	77.80 (107.00)	87.75 (55.23)	0.682^†^
	Total 24 h UFC (> upper limit of normal range/within normal range)	20 (37.0)/34 (63.0)	5 (55.6)/4 (44.4)	7 (38.9)/11 (61.1)	8 (29.6)/19 (70.4)	0.358^‡^
**Hemocytes**	Hemoglobin (g/L)	135.70 (14.62)	142.22 (7.21)	136.67 (17.11)	132.77 (14.28)	0.237^*^
	Red blood cell (10^12^/L)	4.57 (0.48)	4.92 (0.35)	4.56 (0.53)	4.46 (0.44)	0.046^*^
	White blood cell (10^9^/L)	6.00 (1.56)	6.09 (2.22)	5.81 (1.33)	6.10 (1.50)	0.822^*^
	Platelet (10^9^/L)	188.00 (57.05)	186.56 (55.11)	165.06 (51.57)	204.38 (57.71)	0.077^*^
**Liver and kidney function**	ALT (IU/L)	21.00 (12.50)	25.00 (9.00)	17.00 (11.25)	21.00 (17.00)	0.303^†^
	AST (IU/L)	20.52 (7.08)	19.89 (4.43)	19.94 (5.42)	21.11 (8.73)	0.833^*^
	GGT (IU/L)	24.00 (20.50)	24.00 (19.00)	18.00 (19.25)	26.00 (21.25)	0.534^†^
	ALP (IU/L)	76.17 (21.67)	77.56 (29.90)	83.39 (21.37)	70.69 (17.63)	0.158^*^
	Albumin/Globin	1.73 (0.32)	1.92 (0.23)	1.65 (0.31)	1.72 (0.33)	0.102^*^
	Serum creatinine (µmol/L)	75.46 (20.46)	72.62 (19.26)	72.06 (18.35)	78.67 (22.31)	0.521^*^
	eGFR (mL/min/1.73 m^2^)	92.73 (17.65)	111.12 (7.84)	91.83 (14.48)	88.40 (19.01)	0.016^*^
	Serum uric acid (µmol/L)	343.26 (81.95)	328.75 (68.88)	341.61 (75.70)	348.66 (91.16)	0.834^*^
	BUN (mmol/L)	5.11 (1.69)	5.04 (1.56)	4.88 (1.11)	5.29 (2.06)	0.731^*^
**Lipid metabolism**	Triglycerides (mmol/L)	1.55 (0.95)	1.45 (1.04)	1.63 (1.50)	1.51 (1.06)	0.749^†^
	Total cholesterol (mmol/L)	4.22 (0.89)	4.45 (0.82)	4.17 (0.91)	4.18 (0.91)	0.738^*^
	HDL (mmol/L)	1.18 (0.34)	1.21 (0.41)	1.18 (0.29)	1.17 (0.35)	0.959^*^
	LDL (mmol/L)	2.40 (0.73)	2.47 (0.48)	2.37 (0.76)	2.39 (0.79)	0.956^*^
**Glucose metabolism**	Fasting blood glucose before surgery (mmol/L)	7.55 (3.19)	6.70 (2.28)	8.28 (5.32)	7.50 (2.62)	0.398^†^
	HbA1C before surgery (%)	6.60 (1.25)	6.50 (0.88)	7.29 (1.55)	6.50 (1.00)	0.068^†^
	Fasting blood glucose after surgery (mmol/L)	6.36 (1.88)	5.23 (0.88)	6.15 (1.89)	7.08 (2.29)	0.001^†^
	HbA1C after surgery (mmol/L)	6.40 (0.72)	5.70 (0.50)	6.45 (0.55)	6.50 (0.80)	0.001^†^

*Abbreviations*: *PA p*rimary aldosteronism, *BMI* Body mass index, *SBP* Systolic blood pressure, *DM* Diabetes mellitus, *ALD* Aldosterone, *PRA* Plasma renin activity, *ARR* Aldosterone-to-renin ratio, *AT2* Angiotensin II, *K* Potassium, *Na* Sodium, *Cl* Chlorinum, *Ca* Calcium, *Mg* Magnesium, *P* Phosphorus, *PTC8* Plasma total cortisol at 8 am, *PTC0* Plasma total cortisol at 0 am, *ACTH* Adrenocorticotropic hormone, *24 h UFC* 24 h urine free cortisol, *ALT* Alanine aminotransferase, *AST* Aspartate aminotransferase *GGT* Gamma glutamyl transpeptidase, *ALP* Alkaline phosphatase, *eGFR* Estimated glomerular filtration rate, *BUN* Blood urea nitrogen, *HDL* High density lipoprotein, *LDL* Low density lipoprotein, *OGTT0* 0-minute oral glucose tolerance test, *OGTT120* 120-minute oral glucose tolerance test, *HbA1C* glycosylated hemoglobin

*mean (standard deviation). †median (interquartile range). ‡number (percentage)

### Factors associated with the “remission” or “improved” from DM after adrenalectomy in PA patients

The remission and improved groups were considered as a whole group and compared with the unchanged group (Table 2). Variables that significantly differed between the unchanged group and the other two groups by univariate logistic regression analysis was platelet (*P* < 0.05). Then, variables with *P* < 0.10 were entered into the multivariate logistic regression analysis, including duration of hypertension, red blood cell, platelet, alkaline phosphatase, concentration of urinary sodium and 24 h urinary phosphorus. Fasting blood glucose before surgery was also taken into consideration when multivariate logistic regression analysis was performed because it was lower in the “remission” patients. The results showed that PA + DM patients with longer duration of hypertension (odds ratio (OR) = 1.020, 95% confidence interval (CI) 1.002–1.039, *P* = 0.029) were less likely to achieve remission or improvement from DM after adrenalectomy.

**Table 2:** Preoperative factors associated with the “remission” or “improved” DM after adrenalectomy in PA patients

Factors	Univariate logistic regression		Multivariate logistic regression	
	OR (95% CI)	*P* value	OR (95% CI)	*P* value
Age (years)	1.021 (0.969–1.077)	0.434		
Gender (female/male)	1.000 (0.331–3.018)	1.000		
BMI (kg/m^2^)	0.984 (0.825–1.174)	0.857		
Duration of hypertension (months)	1.007 (1.000-1.015)	0.057	1.020 (1.002–1.039)	0.029
Duration of DM (months)	1.005 (0.997–1.013)	0.223		
Order of hypertension and DM (hypertension-DM/DM-hypertension)	0.416 (0.127–1.364)	0.148		
Hemoglobin (g/L)	0.972 (0.935–1.011)	0.155		
Red blood cell (10^12^/L)	0.363 (0.109–1.210)	0.099	0.047 (0-38.342)	0.371
Platelet (10^9^/L)	1.011 (1.000-1.022)	0.046	1.058 (0.935–1.197)	0.370
ALP (IU/L)	0.975 (0.948–1.003)	0.079	1.019 (0.847–1.226)	0.840
Serum creatinine (µmol/L)	1.016 (0.989–1.044)	0.250		
eGFR (mL/min/1.73 m^2^)	0.971 (0.937–1.007)	0.113		
BUN (mmol/L)	1.414 (0.817–1.593)	0.438		
Concentration of urinary K (mmol/L)	1.025 (0.970–1.082)	0.380		
Concentration of urinary Na (mmol/L)	0.982 (0.963–1.002)	0.080	1.055 (0.920–1.209)	0.445
Concentration of urinary Cl (mmol/L)	0.984 (0.964–1.003)	0.106		
Concentration of urinary Ca (mmol/L)	1.017 (0.681–1.521)	0.933		
Concentration of urinary Mg (mmol/L)	1.245 (0.585–2.648)	0.570		
Concentration of urinary P (mmol/L)	0.960 (0.826–1.116)	0.594		
24 h urinary K (mmol/24 h)	1.026 (0.990–1.062)	0.158		
24 h urinary Na (mmol/24 h)	0.997 (0.988–1.007)	0.547		
24 h urinary Cl (mmol/24 h)	0.996 (0.986–1.006)	0.416		
24 h urinary Ca (mmol/24 h)	1.233 (0.794–1.915)	0.351		
24 h urinary Mg (mmol/24 h)	1.860 (0.857–4.034)	0.116		
24 h urinary P (mmol/24 h)	1.199 (0.985–1.460)	0.071	1.036 (0.622–1.725)	0.892
ALD (lying position) (ng/dL)	1.044 (0.984–1.108)	0.150		
ALD (standing position) (ng/dL)	1.003 (0.951–1.058)	0.910		
ARR (lying position)	1.001 (0.999–1.002)	0.415		
ARR (standing position)	1.000 (0.999–1.002)	0.380		
PTC8 (Cortisol) (nmol/L)	0.999 (0.995–1.002)	0.446		
PTC0 (Cortisol) (nmol/L)	0.999 (0.989–1.008)	0.769		
ACTH (ng/L)	1.012 (0.985–1.039)	0.385		
Total 24 h UFC (µg/24 h)	1.007 (0.993–1.022)	0.335		
Fasting blood glucose before surgery (mmol/L)	0.940 (0.776–1.138)	0.524	1.338 (0.530–3.377)	0.537
HbA1C before surgery (%)	0.621 (0.350–1.101)	0.103		

*Abbreviations*: *PA* Primary aldosteronism, *BMI* Body mass index, *DM* Diabetes mellitus, *ALD* Aldosterone, *ARR A*ldosterone-to-renin ratio, *K* Potassium, *Na* Sodium, *Cl* Chlorinum, *Ca* Calcium, *Mg* Magnesium, *P* Phosphorus, *PTC8* Plasma total cortisol at 8 am, *PTC0* Plasma total cortisol at 0 am, *ACTH* Adrenocorticotropic hormone, *24 h UFC* 24 h urine free cortisol, *ALP* Alkaline phosphatase, *eGFR* Estimated glomerular filtration rate, *BUN* Blood urea nitrogen, *OR* Odds ratio, *CI* Confidence interval

### Factors associated the “remission” from DM after adrenalectomy in PA patients

We also compared the remission group with the other two groups (Table 3). Univariate logistic regression analysis revealed that age, red blood cell and estimated glomerular filtration rate (eGFR) were associated with the remission from DM. When controlling for fasting blood glucose before surgery and confounders with *P* < 0.10, including age, red blood cell, eGFR, concentration of urinary potassium, concentration of urinary magnesium and 24 h urinary potassium by multivariate logistic regression analysis, we found that higher concentration of urinary magnesium (OR = 108.200, 95% CI 1.526–7671.118, *P* = 0.031) and higher 24 h urinary potassium (OR = 1.215, 95% CI 1.001–1.476, *P* = 0.049) were factors negatively associated with the “remission” from DM after adrenalectomy in PA patients.

**Table 3:** Preoperative factors associated with the “remission” DM after adrenalectomy in PA patients

Factors	Univariate logistic regression		Multivariate logistic regression	
	OR (95% CI)	*P* value	OR (95% CI)	*P* value
Age (years)	1.118 (1.029–1.215)	0.008	0.923 (0.724–1.177)	0.517
Gender (female/male)	2.333 (0.435–12.530)	0.323		
BMI (kg/m^2^)	1.057 (0.829–1.347)	0.656		
Duration of hypertension (months)	1.007 (0.997–1.018)	0.180		
Duration of DM (months)	1.012 (0.996–1.028)	0.144		
Order of hypertension and DM (hypertension-DM/DM-hypertension)	0.571 (0.106–3.095)	0.516		
Hemoglobin (g/L)	0.960 (0.909–1.014)	0.148		
Red blood cell (10^12^/L)	0.143 (0.025–0.799)	0.027	0.002 (0-5.368)	0.123
Platelet (10^9^/L)	1.001 (0.988–1.013)	0.933		
ALP (IU/L)	0.996 (0.964–1.030)	0.832		
Serum creatinine (µmol/L)	1.009 (0.972–1.046)	0.646		
eGFR (mL/min/1.73 m^2^)	0.862 (0.761–0.976)	0.019	0.682 (0.417–1.115)	0.127
BUN (mmol/L)	1.032 (0.652–1.634)	0.894		
Concentration of urinary K (mmol/L)	1.085 (0.985–1.195)	0.098	1.150 (0.949–1.393)	0.154
Concentration of urinary Na (mmol/L)	0.994 (0.973–1.015)	0.573		
Concentration of urinary Cl (mmol/L)	1.001 (0.978–1.025)	0.912		
Concentration of urinary Ca (mmol/L)	1.541 (0.738–3.219)	0.249		
Concentration of urinary Mg (mmol/L)	5.103 (0.835–31.172)	0.078	108.200 (1.526-7671.118)	0.031
Concentration of urinary P (mmol/L)	0.942 (0.796–1.115)	0.487		
24 h urinary K (mmol/24 h)	1.083 (0.995–1.179)	0.064	1.215 (1.001–1.476)	0.049
24 h urinary Na (mmol/24 h)	1.010 (0.994–1.026)	0.233		
24 h urinary Cl (mmol/24 h)	1.011 (0.993–1.030)	0.223		
24 h urinary Ca (mmol/24 h)	1.385 (0.768–2.497)	0.279		
24 h urinary Mg (mmol/24 h)	2.738 (0.795–9.431)	0.110		
24 h urinary P (mmol/24 h)	1.080 (0.880–1.326)	0.461		
ALD (lying position) (ng/dL)	0.991 (0.927–1.060)	0.794		
ALD (standing position) (ng/dL)	0.996 (0.933–1.063)	0.895		
ARR (lying position)	1.000 (0.998–1.001)	0.680		
ARR (standing position)	1.000 (0.999–1.001)	0.877		
PTC8 (Cortisol) (nmol/L)	1.001 (0.997–1.006)	0.555		
PTC0 (Cortisol) (nmol/L)	1.022 (0.991–1.053)	0.162		
ACTH (ng/L)	1.007 (0.967–1.049)	0.729		
Total 24 h UFC (µg/24 h)	1.005 (0.985–1.025)	0.653		
Fasting blood glucose before surgery (mmol/L)	1.173 (0.854–1.611)	0.325	1.419 (0.656–3.066)	0.374
HbA1C before surgery (%)	0.855 (0.488–1.497)	0.583		

*Abbreviations*: *PA P*rimary aldosteronism, *BMI* Body mass index, *DM* Diabetes mellitus, *ALD* Aldosterone, *ARR* Aldosterone-to-renin ratio, *K* Potassium, *Na* Sodium, *Cl* Chlorinum, *Ca* Calcium, *Mg* Magnesium, *P* Phosphorus, *PTC8* Plasma total cortisol at 8 am, *PTC0* Plasma total cortisol at 0 am, *ACTH* Adrenocorticotropic hormone, *24 h UFC* 24 h urine free cortisol, *ALP* Alkaline phosphatase, *eGFR* Estimated glomerular filtration rate, *BUN* Blood urea nitrogen, *OR* Odds ratio, *CI* Confidence interval

## Discussion

Some studies have analyzed the changes in blood glucose and insulin sensitivity in PA patients after adrenalectomy. Most studies found that blood glucose did not decrease [4, 9, 10]. However, Giacchetti et al. found that the blood glucose level at 2 h after the oral glucose tolerance test dropped in the case of an aldosterone-producing adenoma instead of idiopathic hyperaldosteronism [15]. It was also reported that insulin sensitivity increased after adrenalectomy [10]. The differences between these studies might be attributed to the small sample sizes and the heterogeneity between studies. Additionally, they did not find factors affecting blood glucose after adrenalectomy. In contrast, our study, which had the largest sample size, followed up the DM conditions after adrenalectomy in PA patients. We also analyzed which pre-adrenalectomy factors were associated with the outcome of DM after adrenalectomy.

Our study found that 16.7% of PA + DM patients have achieved remission from DM, and 33.3% of patients were considered “improved” from DM after adrenalectomy, which emphasized the importance of the early identification of PA in DM patients who need adrenalectomy to achieve remission or improvement from DM. Similarly, some studies reported that fasting plasma glucose decreased and insulin sensitivity increased after adrenalectomy [9, 10, 16]. First, they hypothesized that the high blood aldosterone level could induce inflammation and an oxidative stress reaction, which caused injury to the pancreatic beta-cells, followed by reduced insulin secretion. Second, mineralocorticoid receptors have a high affinity for glucocorticoids, which lead to inflammation and insulin resistance through their activation [17]. Third, Adler et al. found that PA patients underwent adrenalectomy or mineralocorticoid receptor antagonist showed the decrease of insulin clearance, which was not due to alterations in creatinine clearance or plasma cortisol [6]. Fourth, aldosterone could increase hepatic glucose production via increased glucose-6-phosphatase and fructose-1,6-bisphosphatase and phosphoenolpyruvate carboxykinase [18]. However, the aldosterone levels of the remission or improvement group and unchanged group were not significantly different in our study. The reason for this may be that the aldosterone levels fluctuated widely before surgery, which may cover up the difference of aldosterone between two groups, especially with a small sample size. In addition, excessive excretion of cortisol may also result in DM in PA patients. Arlt et al. reported that urinary excretion of cortisol and total glucocorticoid level were higher in PA patients compared to those in healthy people [19]. In the present study, we found that both the total 24 h urine free cortisol (UFC) and the percentage of total 24 h UFC higher than the upper limit of normal range (127.55 µg/24 h) were not significantly different between three groups. However, the total 24 h UFC was the highest in the unchanged group, followed by the improved group and the remission group. It indicated that the cortisol level in urine might be associated with the outcome of DM in PA patients after adrenalectomy if sample size of the study was high.

Apart from high blood aldosterone, low blood potassium was another typical feature of PA. Our study revealed that higher 24 h urinary potassium (K) was a factor negatively associated with the “remission” from DM after adrenalectomy in PA patients. However, the blood potassium was not statistically different between three groups. The reason for this phenomenon might be that PA patients would receive oral supplement of potassium before preoperative examination, while low blood potassium induced by high blood aldosterone had lasted for a long time. Some studies reported that there was an inverse relationship between blood potassium level and risk of DM [20, 21]. Rowe and Sagild et al. reported that potassium depletion led to impaired insulin secretion and impaired glucose tolerance [22, 23]. Additionally, Gorden et al. found that those patients with hypokalemia had a higher proportion of circulating proinsulin-like component in insulin secretion [24]. Proinsulin is less biologically active than insulin, resulting in higher serum glucose concentrations. In vitro, hypokalemia prevented the closure of K channels on pancreaticβ-cells and led to decreased insulin secretion [25].

Our study found that the duration of hypertension was much longer in the unchanged group, indicating that a longer duration of hypertension prevented the remission or improvement from DM after adrenalectomy in PA patients. However, the remission or improvement from hypertension did not influence the remission or improvement from DM after adrenalectomy. Although PA can affect patients at any age, studies have reported that PA was commonly diagnosed approximately 50 years old, which indicated the late recognition of PA and its negative effects on DM [26]. Patients with hypertension were found to be 2.5 times more likely to get DM than those with normal blood pressure. Uncontrolled high blood pressure was positively correlated with new-onset type 2 DM [27]. The possible mechanism may be that long-term hypertension-induced vascular endothelium impairment exacerbates insulin resistance by limiting the delivery of glucose to target tissues [28]. Furthermore, patients with uncontrolled hypertension may produce higher amounts of pro-inflammatory cytokines and superoxide anions, which are known to have higher risk of DM [29]. Recent studies also found a relationship between gut microbiota, hypertension and DM. Hypertension may disturb the metabolism of arachidonic acid in gut microbiota, which could play an important anti-inflammatory role [29].

Multivariate logistic regression analyses revealed that higher 24 h urinary magnesium prevented the remission from DM after adrenalectomy. The possible mechanism may be associated with the expansion of the extravascular space, which prevents the reabsorption of magnesium in the proximal tubules, resulting in their excessive excretion from urine [30]. To maintain the blood magnesium level, increasingly more magnesium was released from cells into the blood, leading to intracellular magnesium deficiency [31]. The relationship between magnesium deficiency and type 2 DM has received much attention [32]. Although our study showed that PA patients whose DM did not resolve after adrenalectomy excreted more 24 h urinary magnesium, their serum magnesium was not significantly different when compared with those who achieved remission from DM. The possible reason may be that the total serum magnesium we measured could not represent the intracellular magnesium level, which was shown to be more important in the development of DM. Uğurlu et al. reported that when the cutoff value of intraerythrocyte magnesium was estimated as 4.21 mg/dL, the percentage of low intraerythrocyte magnesium level was higher in DM patients than that in healthy controls [33]. Magnesium played an important role in regulating insulin signals because of its existence in many enzymatic reactions. Chronic magnesium deficiency could reduce intracellular glucose utilization [34]. On the other hand, magnesium deficiency may increase oxidative stress and inflammation, and thus decrease insulin sensitivity [35]. It has been shown that dietary magnesium deficiency may contribute to DM [32]. However, the effects of magnesium supplementation on DM are controversial, where some benefits have been found in several, but not in all, clinical studies [32].

There were some limitations to the study. First, the sample size was small, and the age was statistically lower in remission group, all which may cause biases and affect the statistical analysis. Second, this was a retrospective study, which may cause biases when analyzing the factors affecting the outcome of DM after adrenalectomy. More prospective randomized studies are needed to verify whether adrenalectomy could improve glucose metabolism and which factors are associated with the blood glucose after adrenalectomy.

## Conclusions

Adrenalectomy was beneficial for the remission and improvement from DM in the half of PA patients with DM. Longer duration of hypertension, higher concentration of urinary magnesium and higher 24 h urinary potassium may prevent the remission and improvement from DM after adrenalectomy in PA patients. Examination of urinary electrolyte could be considered in PA patients with DM for predicting the outcome of DM after adrenalectomy.

## Supplementary Information

**Additional file 1:** **Table 1.** The drugs used to treat diabetes mellitus before and after adrenalectomy.

## Data Availability

The datasets generated and/or analysed during the current study are not publicly available because they are the part of the whole database of primary aldosteronism in our hospital, but they are available from the corresponding author on reasonable request.

## Abbreviations

PA: Primary aldosteronism
DM: Diabetes mellitus
ARR: Aldosterone-to-renin ratio
BMI: Body mass index
HbA1C: Glycosylated hemoglobin
ANOVA: Analysis of variance
OR: Odds ratio
CI: Confidence interval
eGFR: Estimated glomerular filtration rate
